# Synthesis, characterization, monolayer assembly and 2D lanthanide coordination of a linear terphenyl-di(propiolonitrile) linker on Ag(111)

**DOI:** 10.3762/bjnano.6.31

**Published:** 2015-01-29

**Authors:** Zhi Chen, Svetlana Klyatskaya, José I Urgel, David Écija, Olaf Fuhr, Willi Auwärter, Johannes V Barth, Mario Ruben

**Affiliations:** 1 Institute of Nanotechnology (INT), Karlsruhe Institute of Technology (KIT), 76344 Eggenstein-Leopoldshafen, Germany; 2 Physik Department E20, Technische Universität München, 85748 Garching, Germany; 3 IMDEA Nanoscience, 28049, Madrid, Spain; 4 Karlsruhe Nano Micro Facility (KNMF), Karlsruhe Institute of Technology (KIT), 76344 Eggenstein-Leopoldshafen, Germany; 5 Universite de Strasbourg, Institut de Physique et de Chimie des Materiaux de Strasbourg, CNRS UMP 7504, 23 Rue du Loess, 67034 Strasbourg Cedex 2, France

**Keywords:** di(propiolonitrile) linker, lanthanides, metal–organic networks, molecular self-assembly, organic monolayers, single crystal X-ray diffraction analysis, UHV-STM

## Abstract

As a continuation of our work employing polyphenylene-dicarbonitrile molecules and in particular the terphenyl derivative **1** (TDCN), we have synthesized a novel ditopic terphenyl-4,4"-di(propiolonitrile) (**2**) linker for the self-assembly of organic monolayers and metal coordination at interfaces. The structure of the organic linker **2** was confirmed by single crystal X-ray diffraction analysis (XRD). On the densely packed Ag(111) surface, the terphenyl-4,4"-di(propiolonitrile) linkers self-assemble in a regular, molecular chevron arrangement exhibiting a Moiré pattern. After the exposure of the molecular monolayer to a beam of Gd atoms, the propiolonitrile groups get readily involved in metal–ligand coordination interactions. Distinct coordination motifs evolve with coordination numbers varying between three and six for the laterally-bound Gd centers. The linker molecules retain an overall flat adsorption geometry. However, only networks with restricted local order were obtained, in marked contrast to previously employed, simpler polyphenylene-dicarbonitrile **1** linkers.

## Introduction

The drive towards miniaturization of modern electronics has led to a growing interest in the development of memory units that can satisfy the ever-growing demand for information storage. In this context, rare-earth elements have been employed for the design of materials with extraordinary magnetic properties [1–2], including single molecular magnets (SMMs) [2–3], which serve as pivotal subunits for modern developments in spintronic devices [4–12].

Moreover, in recent years, significant strides have been made in the understanding and the application of nanofabrication from the "bottom-up" perspective [13–17]. The tailored design, controlled formation, and in-depth characterization of self-assembled, molecular and periodic heterostructures (ranging over several length scales on atomically well-defined surfaces under ultrahigh vacuum (UHV) conditions) have been achieved [13–15,18–19]. More recently, our groups have successfully extended this approach toward the on-surface coordination of f-block organic networks exhibiting five-vertex, Archimedean surface tessellation [20–21]. However, at least for the class of simple polyphenylene dicarbonitrile linkers, NC–Ph*_n_*–CN (*n* = 3, **1**), the nature of the underlying mononuclear five-fold coordination motif is still unclear. It is notably an open question whether the nature of the surface interaction or the steric hindrances of the surface-confined groups are crucial factors favouring the expression of certain coordination motifs.

In this context, the nature of the organic linker molecule seems to play a crucial role in the tuning of the on-surface 2D self-assembly, by means of the intermolecular and substrate-mediated interactions [22–24]. A particular case is represented by molecules that show highly reactive functional units, such as terminal carbon–carbon triple bonds (–C≡CH) [25–32]. Notably, when working on a planar Ag(111) substrate, in addition to the reported butadiyne bridge formation via a homocoupling reaction, a clear tendency toward branching-side reactions involving three and four reacting monomers and leading to markedly reduced chemoselectivity is observed [25–28]. Interestingly, on a Au(111) substrate, the cyclotrimerisation of arylalkynes becomes the dominant reaction pathway with high selectivity [33]. The alkynyl activation leading to C–C coupling has been ascribed to the emergence of a double σ-bridge-bounded acetylene [25–26], or alternatively to the formation of an intermediary π-substrate complex [27–28,34].

The results presented herein focus on the design, synthesis, characterization and 2D Ag(111)-mediated self-assembly of a novel terphenyl-4,4"-di(propiolonitrile) (**2**) linker exhibiting a NC–C≡C–Ph_3_–C≡C–CN structure. Based on previous work in our group employing dicarbonitriles, as well as diacetylenes, the linker **2** carries both a –C≡C– acetylene group and a terminal carbonitrile group (–C≡N). In bulk chemistry, this combination usually yields so-called propiolonitriles as versatile building blocks for highly functionalized derivatives [35–41]. The structure of the organic linker **2** was confirmed by single crystal X-ray diffraction analysis (XRD) along with other standard techniques (Supporting Information File 1).

The results of the surface-confined, molecular self-assembly and the lanthanide coordination reaction were analysed by using low-temperature scanning tunnelling microscopy (STM). The STM investigation of the self-assembly of the organic linker **2** on a Ag(111) surface revealed a densely packed, chevron monolayer exhibiting a Moiré pattern. In contrast, lanthanide coordination of the same ligand **2** with Gd atoms resulted in metal–organic networks with only local order. These latter results differ strongly from previous reports on 2D surface coordination of the related ligand terphenyl-4,4"-dicarbonitrile (**1**) linker by cerium or gadolinium atoms [20–21,42]. This indicates that the preference for the formation of extended metal–organic networks is not primarily a consequence of the geometrical footprint of the endgroups at the surface, but rather a generic property of carbonitrile–Ln coordination.

## Results and Discussion

### Synthesis

**Figure 1:**
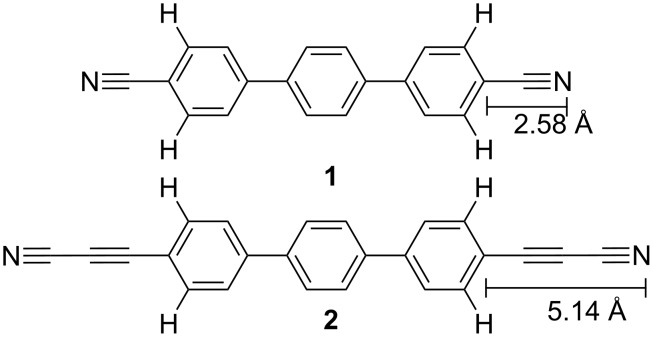
Representation of the structure of the molecular linkers: terphenyl-4,4"-dicarbonitrile (**1**) [24,43], terphenyl-4,4"-di(propiolonitrile) (**2**) showing the increased distance of the coordinating carbonitrile N-donor atom from the sterically hindering α-C–H group at the phenyl ring.

This work compares the 2D self-assembly and the coordination behaviour of two related ligand systems, namely the terphenyl-4,4"-dicarbonitrile (**1**) and the terphenyl-4,4"-di(propiolonitrile) (**2**) linker, whereby the latter has been synthesized and characterized herein (Figure 1). A class of terphenyl-4,4"-dicarbonitrile derivatives, NC–Ph_3_–CN, was intensively studied as a linker in molecular and metal coordination assemblies under 2D confinement [20,22,24,43]. With the goal to achieve increased coordination numbers (7–12) (typically for f-block elements in bulk chemistry [18,20,44]), the linker **2** was designed to reduce the steric repulsion induced by the α-C–H bonds at the terminal phenyl rings and the coordinating donor N-atom of the carbonitrile group. By incorporating the propiolonitrile groups into the terphenyl backbone of NC–C≡C–Ph_3_–C≡C–CN (**2**), the distance between the coordination-active N-atom of the –C≡N group and the adjacent phenyl ring bearing the α-C–H group is consequently increased from 2.58 Å to 5.14 Å. (Figure 1).

**Scheme 1:**
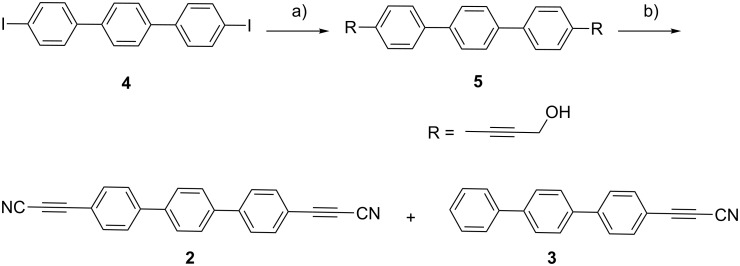
Synthesis of the terphenyl-4,4"-di(propiolonitrile) linker (**2**). Reagents and conditions: a) propargyl alcohol, Pd(PPh_3_)_2_Cl_2_/CuI, pyrrolidine/THF, 60 °C; b) NH_3_–IPA, MgSO_4_, MnO_2_, THF, rt [45].

During the synthesis, the diiodoterphenyl **4** was subjected to a cross-coupling reaction with propargyl alcohol in the presence of catalytic amounts of Pd(II) salts, leading to the formation of the intermediary compound **5**. This compound was subsequently reacted by a tandem manganese dioxide-mediated alcohol oxidation with in situ trapping of the resulting aldehydes with ammonia giving the final product **2** with a overall yield of 18% [45] (Scheme 1).

**Figure 2:**
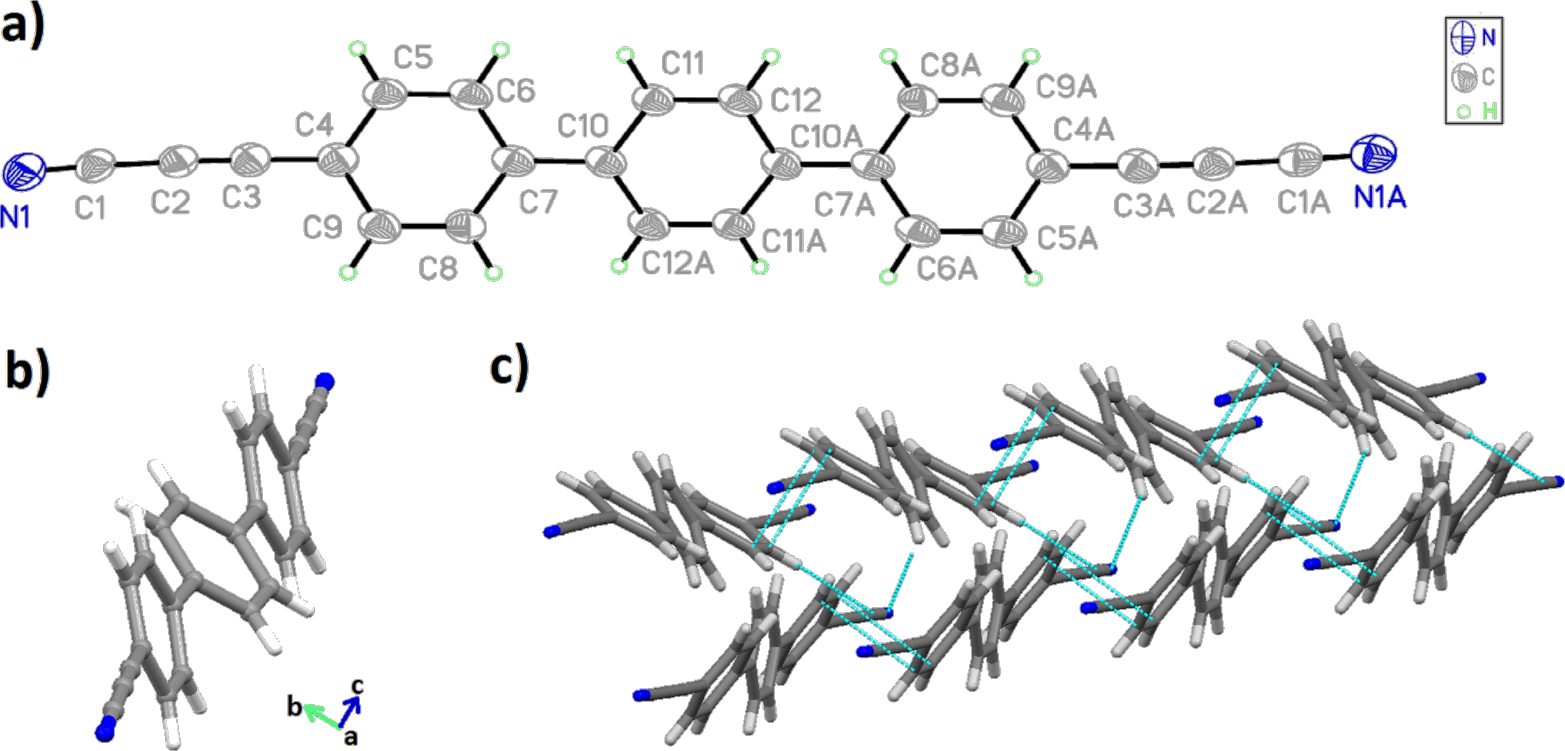
ORTEP plot of compound **2**. Ellipsoids were drawn at a 30% level of probability for all non-hydrogen atoms, indicating the numbering scheme (a); 3D visualization of the molecular conformation (view along direction *a*) (b); and the packing viewed along the b* axis demonstrating the parallel layers arrangement (c)*.*

Additionally, a small amount of a byproduct, identified as terphenyl-4-propiolonitrile (**3**) (Ph_3_–C≡C–C≡N), was separated and revealed to be a thus-far unreported decarboxylation reaction of the propiolonitrile group. The nature of this compound was confirmed by a blind synthesis starting from 4-bromoterphenyl (**6**) following a multistep protocol (Supporting Information File 1, Scheme S1). The nature of both compounds, the di- (**2**) and the mono-substituted linker (**3**), was substantiated by single crystal X-ray structure analysis at 180 K. Compound **2** crystallizes in a monoclinic system with space group *P*2_1_/*n*, while **3** crystallizes in the triclinic system with space group P-1. The anisotropic displacement ellipsoids and atom labelling (ORTEP plots) of compounds **2** and **3** are shown in Figure 2a and Supporting Information File 1, Figure S2, respectively. Selected bond lengths of these molecules are listed in Supporting Information File 1, Tables S1–4.

The visualization of molecule **2** highlights a conformation, in which the central phenyl ring is rotated out of the plane of the two peripheral ones by a dihedral angle of 31.56(5)° (Figure 2b). In comparison, the mono-substituted compound **3** exhibits twist angles of 33° and 10° between phenyl rings A/B and B/C, respectively (Supporting Information File 1, Figure S3). In both cases the molecules arrange within the crystal in layers (Figure 2c and Supporting Information File 1, Figure S3) in an antiparallel organisation of the end standing CN groups by dipole–dipole interactions.

### Formation of the self-assembled monolayer of **2** on Ag(111)

In recent years, systematic studies of the self-assembly behavior of the series of polyphenylene-dicarbonitrile linkers (NC–Ph*_n_*–CN, *n* = 3–6) on the Ag(111) surfaces have been reported demonstrating the controlled formation of highly-ordered monolayers [43,46]. The structural diversity of the formed molecular monolayers was illustrated by (i) a strict dependence on the length of linker molecules resulting in either densely packed chevron patterns (*n* = 3), open rhombic networks (*n* = 4) or complex Kagomé lattices (*n* = 5, 6); (ii) changing the stereochemical position of the coordinating –CN groups leading to higher order complexities with partially systemic behavior [47–51].

**Figure 3:**
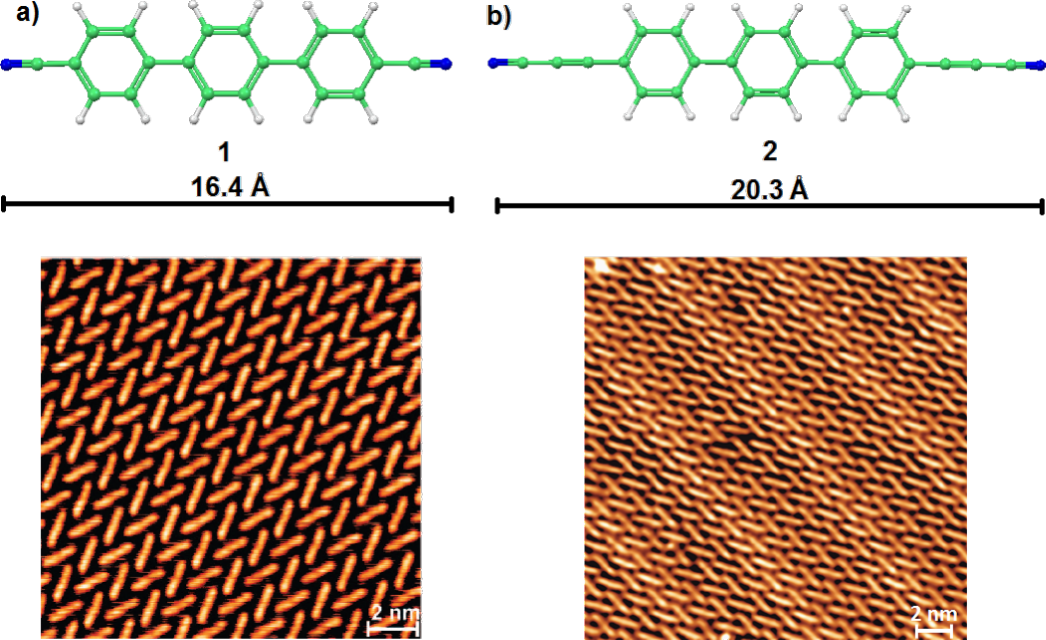
Comparison of the molecular self-assembled monolayers of **1** and **2** on a Ag(111) surface. a) Densely packed chevron layer formed by NC–Ph_3_–CN (**1**), adapted from [43]; b) Densely packed chevron layer formed by the NC–C≡C–Ph_3_–C≡C–CN (**2**) species (data measured at 6 K; image size: 239 × 239 Å^2^; scanning parameters: *V*_bias_ = 0.3 V, *I* = 0.05 nA).

The new linker **2** (NC–C≡C–Ph_3_–C≡C–CN) was deposited by organic molecular beam epitaxy onto an atomically clean and flat Ag(111) surface kept at 300 K. After the deposition, the samples were cooled down to about 6 K for imaging. Similar to earlier studies on the terphenyl-dicarbonitrile analog **1** [43], the individual molecules of NC–C≡C–Ph_3_–C≡C–CN (**2**) were clearly resolved as rod-like protrusions showing a chevron arrangement but now exhibiting an additional Moiré pattern (Figure 3). The latter results from the superposition of the monolayer and substrate symmetries, rotated by an angle showing the very subtle balance between molecule–substrate and molecule–molecule interactions. Occasionally, deviations from molecular linearity as an S-shape of certain protrusions could be identified. Similar to the earlier observations for dicarbonitrile oligophenyls [43,46,52], this contrast can be ascribed to the rotation of the aromatic rings alternatively lifting the left and the right side of a phenyl rings up from the underlying substrate.

It was found that the chevron pattern assembled from **2** is similar to that earlier reported for NC–Ph*_n_*–CN linkers (whereby *n* = 3, **1**) [43], where only two orientations of the molecules with the respect to the substrate within a given domain are present.

**Figure 4:**
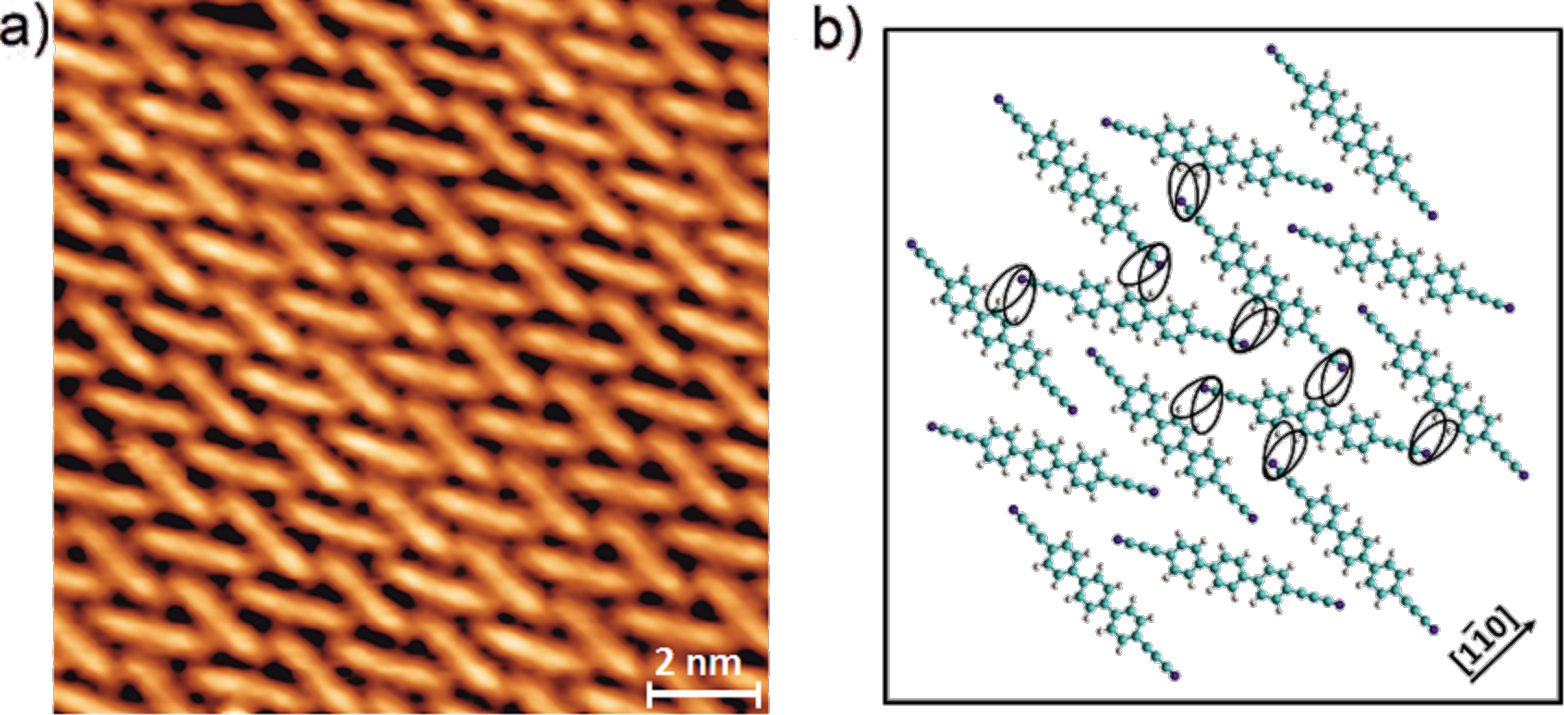
High-resolution STM image showing a) the molecular packing in chevron layers mediated by the propilonitrile end groups of the NC–C≡C–Ph_3_–C≡C–CN (**2**) linker (data measured at 6 K; image size: 119 × 119 Å^2^; scanning parameters: *V*_bias_ = 0.3 V, *I* = 0.05 nA); b) Corresponding model showing the interaction of adjacent propionitrile groups.

The high-resolution STM topograph depicted in Figure 4a clearly indicates that the monolayer pattern is determined by non-covalent interactions between adjacent linkers, in particular electrostatic interactions [43,47,53]. The packing is stabilized by the attractive interaction between the propilonitrile end groups, as proton acceptors, and H atoms of the phenylene rings. This reveals related ordering principles on the terminal alkynes, which is interpreted in terms of a proton acceptor–ring interaction [53].

Figure 4b represents the model based on the averaged bonding distances and angles between adjacent molecules gained from the STM data. The derived organic network model expresses a six-fold symmetry related to the substrate atomic arrangement. Based on the topography and assuming the same size of the molecules as in the gas phase, we obtained an average length of about 0.26 ± 04 nm for the phenylene–N distance, which is slightly shorter than in the earlier reported network of molecule **1** (0.33 ± 03 nm) [43].

### Lanthanide-directed coordination of **2** on Ag(111)

In previous work, regular metallo–supramolecular nanomeshes were obtained on flat Ag(111) surfaces from the exposure of **1**-type NC–Ph*_n_*–CN (*n* = 3, 4, 5, 6) linkers to cobalt atoms [22–23,48], while the use of lanthanide atoms (Ce, Gd) yielded an Archimedean snub square tiling [20–21]. The underlying driving force for the diversity in results is associated with the remarkable coordination reactivity of carbonitrile groups, which are very well known in bulk coordination chemistry.

The linker **2** was deposited by organic molecular beam epitaxy onto an atomically clean and flat Ag(111) surface kept at 300 K, followed by the controlled co-deposition of Gd atoms provided from an electron beam source. The samples were subsequently cooled down to *T* ≈ 6 K for imaging.

**Figure 5:**
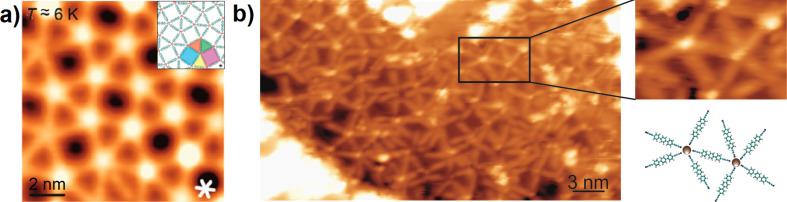
STM image of the lanthanide-directed assembly on Ag(111) for appreciable surface concentration (linker: Ln = 5:2). a) Snub square tiling motif comprised of NC–Ph_3_–CN (**1**) linkers and Gd centers (atomistic model of the snub square Archimedean tessellation of the surface in insert), reprinted with permission from [21], copyright 2014 American Chemical Society; b) irregular metal–organic network comprised of NC–C≡C–Ph_3_–C≡C–CN (**2**) molecules and Gd centers. In inset: model of one of the coordination units presented (the 5-fold linker Gd nonameric unit. Data was obtained at 6 K. Image size: 352 × 195 Å^2^. Scanning parameters: *V*_bias_ = 0.7 V, *I* = 0.1 nA).

In contrast to previous reports on NC–Ph_3_–CN (**1**) (Figure 5a) [20–21], the co-evaporation of Gd atoms with linker **2** resulted in an irregular metal–organic pattern without expression of a translational spatial symmetry. Thus, nodes with variable coordination motifs, including clustering, can be found in the STM topographs (Figure 5b). Consequently, no clear preference of one coordination motif with higher coordination number was encountered, even in the presence of excess Gd (associated with cluster formation as shown by the emergence of white protrusions in Figure 5b). This provides an unintended route towards 2D, randomly reticulated coordination networks, which is in line with the usage of linear and nonlinear dicarbonitrile linkers as recently reported [48,50].

A model for a five-fold coordination vertex and detailed views of the respective STM data are reproduced in Figure 5b. Despite serious efforts, only surface-confined networks with limited length scales could be obtained. Obviously, the expression of regular metallo–supramolecular nanostructures or layers requires a careful balance of surface bonding, mobility and lateral interactions between metal centers and linkers [54]. We attribute the observed, reduced order to the high reactivity of the –C≡C– bonds in propiolonitrile groups. From bulk chemistry, it is well known that the activation of the acetylene group by noble metal substrates can occur [35–41]. This was demonstrated by on-surface homo-coupling of alkynes on planar surfaces with a clear tendency towards branching side reactions [25–28,34]. Although in the presented work we could not deduce any changes of the –C≡C– bonds from the STM investigations, we attribute the hampering of the expression of a regular network to the presence of active –C≡C– bonds close to the coordinating CN units.

## Conclusion

The self-assembly of the new terphenyl-4,4”-di(propiolonitrile) (**2**) linker on the Ag(111) surface leads to a densely packed monolayer with chevron arrangement exhibiting a Moiré pattern. Gd-directed assembly resulted in an irregular metal–organic pattern with variable coordination motifs, but without any evidence of coordination numbers higher than five. The preference for the established mononuclear five-fold nodes, identified previously for the related class of linkers of type **1**, seems thus a generic property of the 2D carbonitrile–Ln coordination. Obviously, the high reactivity of the –C≡C– bonds in the propiolonitrile groups prevented the surface-confined molecular system from formation of regular metal–organic nanostructures or layers, instead resulting in reticulated Ln, organic networks with only local order. Our results highlight the paramount importance of the nature of the coordinating end groups for the surface-confined lanthanide coordination chemistry in attempts to design molecular architectures incorporating the sophisticated properties of f-elements [55].

## Experimental

### STM measurements

The STM measurements were performed using a CreaTec low temperature STM (LT-STM). The base pressure of the ultrahigh vacuum system was below 2 × 10^−10^ mbar.

The Ag(111) substrate was prepared using standard cycles of Ar^+^ sputtering (800 eV) and subsequent annealing at 723 K for 10 min. All STM images were taken in constant-current mode with an electrochemically etched tungsten tip.

The supramolecular networks based on Gd–ligand coordination motifs described in the manuscript were fabricated in a two-step process:

1. The molecular linkers NC–C≡C–Ph_3_–C≡C–CN (**2**) were deposited from a quartz crucible held at *T* = 479 K by organic molecular beam epitaxy (OMBE) onto a clean Ag(111) crystal held at ≈300 K.
2. Subsequently, Gd atoms were sublimated by means of electron beam evaporation from an outgassed Gd rod (99.9%, MaTecK GmbH, 52428 Jülich, Germany) onto the sample held at ≈300 K.

### X-ray crystallography

Crystals suitable for single crystal X-ray diffraction were obtained by slow diffusion of hexane into a 1,4-dioxane solution of **2** and by slow diffusion of hexane into a solution of **3** in the dichloromethane. Crystals were then selected in perfluoroalkyl ether oil. Single crystal X-ray diffraction data of compounds **2** and **3** were collected on a STOE IPDS II diffractometer with graphite monochromatic Mo Kα radiation (0.71073 Å) at 180 K.

Data were corrected for Lorentz and polarisation effects. Interframe scaling was performed with the program LANA. The structures were solved by direct methods (SHELX-97) [56]. Refinement was performed with anisotropic temperature factors for all non-hydrogen atoms. Crystal data and the results of the refinement are collected in Supporting Information File 1, Tables S1–4. Molecular diagrams were prepared using Diamond software [57].

CCDC-1026443 (**2**) and CCDC-1006987 (**3**) contain the supplementary reference crystallographic data for this paper. These data can be obtained free of charge at http://www.ccdc.cam.ac.uk/conts/retrieving.html (or from the Cambridge Crystallographic Data Centre, 12 Union Road, Cambridge CB2 1EZ, UK; fax: +44 1223/336-033; Email: deposit@ccdc.cam.ac.uk).

### General synthesis remarks

Reactions requiring an inert gas atmosphere were conducted under argon, and the glassware was oven dried (140 °C). All reagents were purchased from commercial sources and used as received. Compound **4** was prepared according to previous literature [58].

^1^H NMR and ^13^C NMR spectra were recorded on a Bruker DRX 500 spectrometer. The chemical shifts are given in ppm and are referenced to residual proton resonances of the solvents. The mass spectroscopic data were acquired with a Voyager-DE PRO Bio spectrometry work station for MALDI–ToF. MALDI spectra were measured with no additional matrix compound other than the sample itself. Elemental analysis of carbon, hydrogen, and nitrogen were carried out in a Vario Micro Cube. Infrared spectra were measured in KBr pellets (MAGNA FTIR 750, Nicolet) in the 4000–400 cm^−1^ region.

### 3,3'-([1,1':4',1''-terphenyl]-4,4''-diyl)bis(prop-2-yn-1-ol) (**5**)

Under an argon atmosphere 4,4''-diiodo-1,1':4',1''-terphenyl (**4**, 192 mg, 1.0 mmol), prop-2-yn-1-ol (140 mg, 2.5 mmol), Pd(PPh_3_)_2_Cl_2_ (40 mg), CuI (20 mg) were added to a mixture of 10 mL pyrrolidine and 10 mL THF and heated at 60 °C for 36 h. Next, hexane (50 mL) was added and the residue was filtered off and dissolved in THF. The solution was chromatographed on silica gel using dichloromethane/ethyl acetate 5:1 as eluent with a short column, affording 240 mg of **5** as yellow solid (yield 71%). ^1^H NMR (500 MHz, DMSO-*d*_6_) δ/ppm 4.34 (d, *J* = 5.96 Hz, 4H, –CH_2_–), 5.37 (t, *J* = 5.96, 5.96 Hz, 2H, –OH), 7.54 (d, *J* = 8.37 Hz, 4H, Ar–H), 7.77 (d, *J* = 8.39 Hz, 4H, Ar–H), 7.82 (s, 4H, Ar–H); ^13^C NMR (126 MHz, DMSO-*d*_6_) δ/ppm 139.21, 138.43, 131.88, 127.20, 126.74, 121.62, 90.79, 83.39, 49.48; IR (KBr, cm^−1^): 2184 (C≡C); MALDI–ToF (*m*/*z*): [M]^+^ calcd for C_24_H_18_O_2_, 338.1; found, 338.0.

### 3,3'-([1,1':4',1''-terphenyl]-4,4''-diyl)dipropiolonitrile (**2**)

Following [45] a 2 M solution of ammonia in 2-propanol (1.8 mL, 3.2 mmol) and anhydrous magnesium sulfate (1.5 g, 12.8 mmol) were added to a stirred solution of compound **5** (203 mg, 0.6 mmol) in THF (20 mL). Then, activated manganese dioxide (1.1 g, 12.8 mmol) was added. The resulting mixture was stirred at room temperature for 2 h and then diluted with dichloromethane (20 mL). The mixture was filtered through Celite, washed well with dichloromethane, and the combined filtrates were concentrated in vacuum. The residue was purified by column chromatography on silica gel (hexane/dichloromethane 2:1) affording 51 mg of **2** as light yellow solid (yield 26%). ^1^H NMR (500 MHz, CDCl_3_) δ/ppm 7.69–7.75 (m, 12H); ^13^C NMR (126 MHz, CDCl_3_) δ/ppm 143.69, 139.46, 134.11, 127.85, 127.41, 116.60, 105.54, 82.86, 63.95; IR (KBr, cm^−1^): 2260 (C≡N), 2141, (C≡C); MALDI–ToF (*m*/*z*): [M]^+^ calcd for C_24_H_12_N_2_, 328.1; found, 328.1; Anal. calcd for C_24_H_12_N_2_: C, 87.79; H, 3.68; N, 8.53; found: C, 87.63; H, 3.45; N 8.81.

Additionally, 10 mg of a white solid was separated. The analytical data were identical to terphenyl-4-propiolonitrile (**3**) prepared by the blind synthesis (Supporting Information File 1).

## Supporting Information

File 1 Additional experimental data.
