# Supplementary material for: Synthesis, characterization, monolayer assembly and 2D lanthanide coordination of a linear terphenyl-di(propiolonitrile) linker on Ag(111)
Source: Beilstein J Nanotechnol. 2015 Jan 29;6:327–35. doi: 10.3762/bjnano.6.31 (PMC4362489; doi:10.3762/bjnano.6.31)
Supplement: File 1 — Additional experimental data. [file Beilstein_J_Nanotechnol-06-327-s001.pdf]

Supporting Information

for

Synthesis, characterization, monolayer assembly and 2D lanthanide coordination of a linear terphenyl-di(propiolonitrile) linker on Ag(111)

Zhi Chen[1], Svetlana Klyatskaya*[1], José I. Urgel[2], David Écija[2,3], Olaf Fuhr[1,4], Willi Auwärter[2], Johannes V. Barth*[2] and Mario Ruben*[1,5]

Address: [1]Institute of Nanotechnology (INT), Karlsruhe Institute of Technology (KIT), 76344 Eggenstein-Leopoldshafen, Germany, [2]Physik Department E20, Technische Universität München, 85748 Garching, Germany, [3] IMDEA Nanoscience, 28049, Madrid, Spain, [4]Karlsruhe Nano Micro Facility (KNMF), Karlsruhe Institute of Technology (KIT), 76344 Eggenstein-Leopoldshafen, Germany and [5]Universite de Strasbourg, Institut de Physique et de Chimie des Materiaux de Strasbourg, CNRS UMP 7504, 23 Rue du Loess, 67034 Strasbourg Cedex 2, France

Email: Svetlana Klyatskaya - svetlana.klyatskaya@kit.edu; Johannes V. Barth - jvb@tum.de; Mario Ruben - mario.ruben@kit.edu

* Corresponding author

Synthesis of compound 3

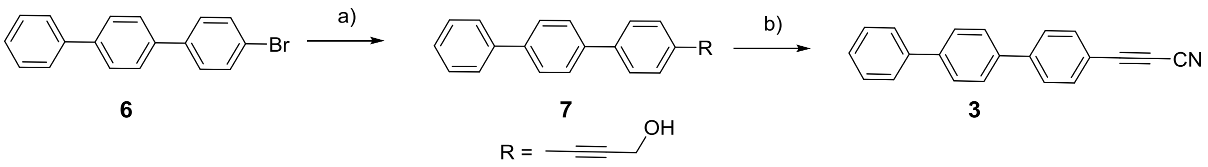

Scheme S1: Synthesis of compound **3**. Reagents and Conditions: a) propargyl alcohol, $Pd(PPh_3)_2Cl_2$/CuI, pyrrolidine/THF, 60 °C; b) NH_3-IPA, $MgSO_4$, MnO_2, THF, rt [1].

The monotopic molecular linker **3**, with only one incorporated propiolonitrile group, was synthesized from 4-bromoterphenyl **6** following the multistep protocol (Scheme S1), which involves at first the cross-coupling reaction with propargyl alcohol in the presence of the catalytic amount of Pd(II) salt. Secondly, **7** was converted into the corresponding nitrile **3** by treatment with ammonia in 2-propanol (IPA) and THF containing magnesium sulfate and manganese dioxide at room temperature for 2 hours.

3-([1,1':4',1''-terphenyl]-4-yl)prop-2-yn-1-ol (7)

4-bromo-1,1':4',1''-terphenyl (**6**, 412 mg, 1.5 mmol), prop-2-yn-1-ol (126 mg, 2.25 mmol), $Pd(PPh_3)_2Cl_2$ (50 mg), CuI (30 mg), PPh_3 (40 mg) were added to a mixture of 30 mL pyrrolidine and 15 mL THF and heated under reflux for 10 h under an argon atmosphere [2]. Hexane (50 mL) was added, and the residue was filtered off and dissolved in dichloromethane. The solution was chromatographed on silica gel (hexane/dichloromethane 1:1) affording 120 mg of **7** as white needle-like crystals (yield 28%).

^{1}H NMR (500 MHz, $CDCl_3$): δ/ppm 4.56 (d, 2H, $-CH_2-$), 7.36-7.74 (m, 13H, Ar-H). ^{13}C NMR (126 MHz, $CDCl_3$): δ/ppm 140.75, 140.58, 140.54, 139.12, 132.20, 128.87, 127.61, 127.49, 127.39, 127.06, 126.89, 121.49, 87.90, 85.66, 51.79. IR (KBr, cm^{-1}): 2184(C≡C). MALDI ToF calculated for $C_{21}H_{16}O$: M^+, 284.1; found: *m*/*z*, 284.0.

3-([1,1':4',1"-terphenyl]-4-yl)propiolonitrile (3)

Following [1], a 2 M solution of ammonia in 2-propanol (0.8 mL, 1.6 mmol) and anhydrous magnesium sulfate (768 mg, 6.4 mmol) were added to a stirred solution of compound **7** (114 mg, 0.4 mmol) in THF (20 mL). Then, activated manganese dioxide (557 mg, 6.4 mmol) was added. The resulting mixture was stirred at room temperature for 2 h and then diluted with dichloromethane (20 mL). The mixture was filtered through Celite, washed well with dichloromethane, and the combined filtrates were concentrated in vacuum. The residue was purified by column chromatography on silica gel (hexane/dichloromethane 2:1) affording light yellow solid compound **3** (37 mg, 33%).

^{1}H NMR (500 MHz, $CDCl_3$): δ/ppm 7.41 (t, *J* = 7.38, 7.38 Hz, 1H, Ar-H), 7.50 (t, *J* = 7.64, 7.64 Hz, 2H, Ar-H), 7.64–7.74 (m, 10H, Ar-H). ^{13}C NMR (126 MHz, $CDCl_3$): δ/ppm 144.19, 141.44, 140.25, 138.16, 134.06, 128.93, 127.79, 127.72, 127.56, 127.34, 127.08, 116.16, 105.61, 83.11, 63.79. IR (KBr, cm^{-1}): 2263 (C≡N), 2143(C≡C). MALDI ToF calculated for $C_{21}H_{13}N$: M^+, 279.1; found: *m*/*z*, 279.0. Elemental analysis calculated (%) for $C_{21}H_{13}N$: C 90.29, H 4.69, N 5.01; found: C 90.24, H 4.91, N 5.07.

Crystallographic study of the compound 2

Crystals of compound **2** suitable for XRD were grown from dichloromethane and dioxane solvents mixture. Compound **2** crystallizes in the monoclinic system with space group $P2_1/n$. The asymmetric unit consists of a half of the crystallographically independent molecule of compound **2** (Table S1). The twist angles between phenyl rings are 31° (Figure S1a, b). The long axis of the molecule is distorted such that carbon atoms connecting the phenyl rings deviate from the straight line connecting the terminal nitrogen atoms. Similar distortions are quite common for molecules with aromatic rings connected in para position [4,5]. Within the solid phase of compound **2**, the shortest CH⋯N≡C hydrogen bond distances are 2.89 Å and 2.70 Å (N⋯H separation). The chain formation of NC–C≡C–Ph_3–C≡C–CN results from H bonding and π-stacking. The different layers stack in molecular columns oriented along the *b*-axis with identical arrangement of the individual moieties resulting in a π-stacking distance of 3.39 Å as can be seen in Figure S1b, typically appearing for π-stacking.

a)

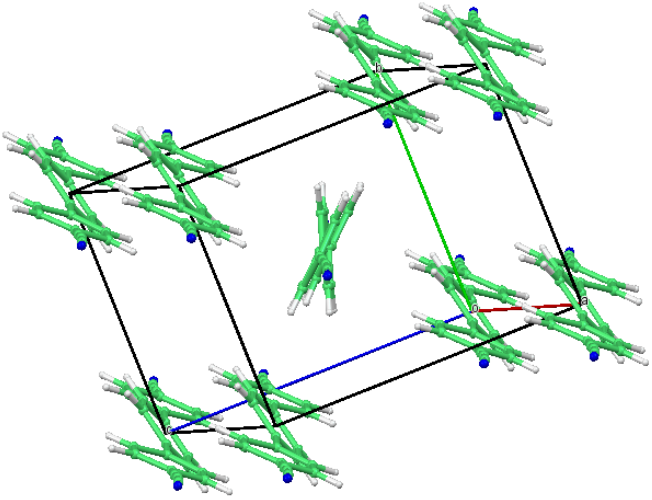

b)

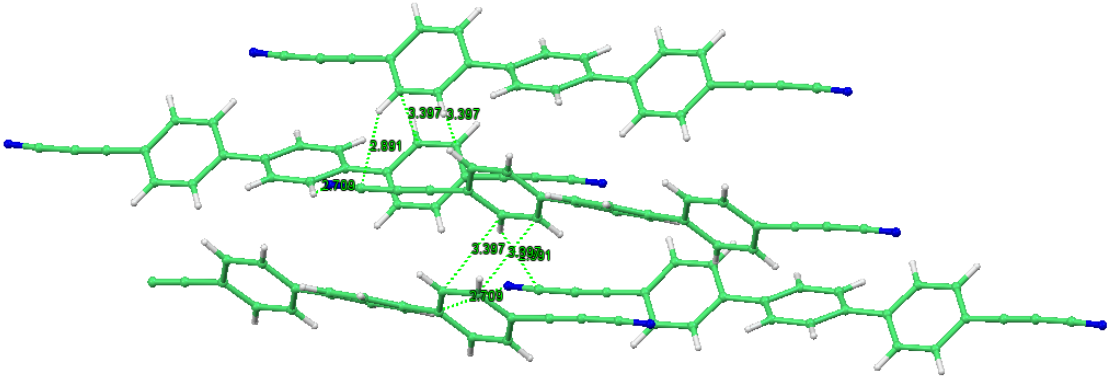

Figure S1: The crystal structure of compound **2** as obtained by X-ray structure analysis. a) Molecules arrange in parallel layers. Propiolonitrile groups acquire an antiparallel ordering motif. One H bond of the N⋯CH type is present (b). All distances are given in Å.

Table S1: Crystal data and structure refinement for **2**.

Compound	**2**
Empirical formula	$C_{24}H_{12}N_2$
Formula weight	328.36
Temperature/K	180.15
λ/Å	0.71073
Crystal system	monoclinic

Space group	$P2_1/n$
a/Å	9.9017(17)
b/Å	8.3916(18)
c/Å	10.6222(19)
α/°	90
β/°	110.809(13)
γ/°	90
Volume/Å^3	825.0(3)
Z	2
ρ_{calc} mg/mm^3	1.322
μ/mm^{-1}	0.078
F(000)	340.0
Crystal size/mm^3	0.32 × 0.1 × 0.08
2θ range for data collection/°	6.356 to 51.458
Reflections collected	3271
Independent reflections	1537 [R_{int} = 0.1376, R_{sigma} = 0.1148]
Data/restraints/parameters	1537/0/119
Goodness-of-fit on F^2	0.926
Final R indexes [I >= 2σ (I)]	R_1 = 0.0791, wR_2 = 0.1947
Final R indexes [all data]	R_1 = 0.1448, wR_2 = 0.2349
Largest diff. peak/hole / e Å^{-3}	0.21/−0.26

Table S2: Selected bond lengths (Å) in compound **2**.

N1–C1	1.161(5)	C7–C8	1.401(5)
C1–C2	1.365(6)	C7–C10	1.471(5)
C2–C3	1.189(5)	C8–C9	1.369(5)
C3–C4	1.427(6)	C10–C11	1.391(5)
C4–C5	1.401(5)	C10–C12^1	1.402(5)
C4–C9	1.399(5)	C11–C12	1.368(5)
C5–C6	1.375(5)	C12–C10^1	1.402(5)
C6–C7	1.392(5)		

Crystallographic study of the mono-topic by-product 3

Crystals of compound **3** suitable for XRD were grown from dichloromethane and hexane solvents mixture. Compound **3** crystallizes in the triclinic system with space group *P*-1. The asymmetric unit consists of three crystallographically independent molecules of compound **3** (Table S3). All three molecules exhibit screw-like structures resulting from a gradual rotation of the phenyl rings. In contrast to the ditopic molecule **2**, the twist angles between phenyl rings A/B and B/C are 33° and 10° respectively. The twist angles between phenyl rings in the other two independent molecules are average of 35° (Figure S3). Each nitrogen atom establishes two CH···N hydrogen bonds, one connecting to the neighboring molecule within the layer (Figure S3) with a N···H separation of 2.56 Å and one connecting to a molecule of the next layer with the separation of 2.62 Å. As can be seen in Figure S3, a layer-by-layer arrangement is present in the crystal phase, too, but no direct stacking of identically oriented molecules as in the case of compound **2** takes place.

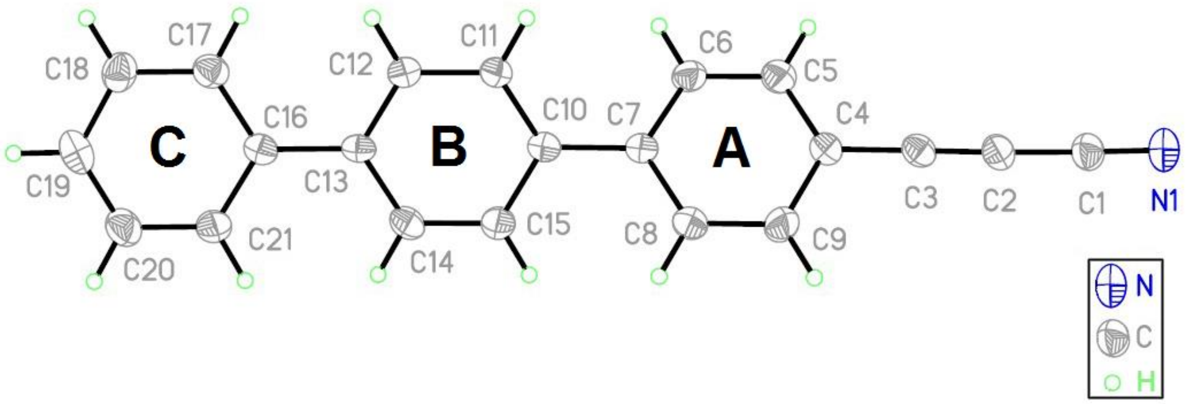

Figure S2: ORTEP plot of compounds **3** with ellipsoids drawn at 30% level of probability for all non-hydrogen atoms, indicating the numbering scheme.

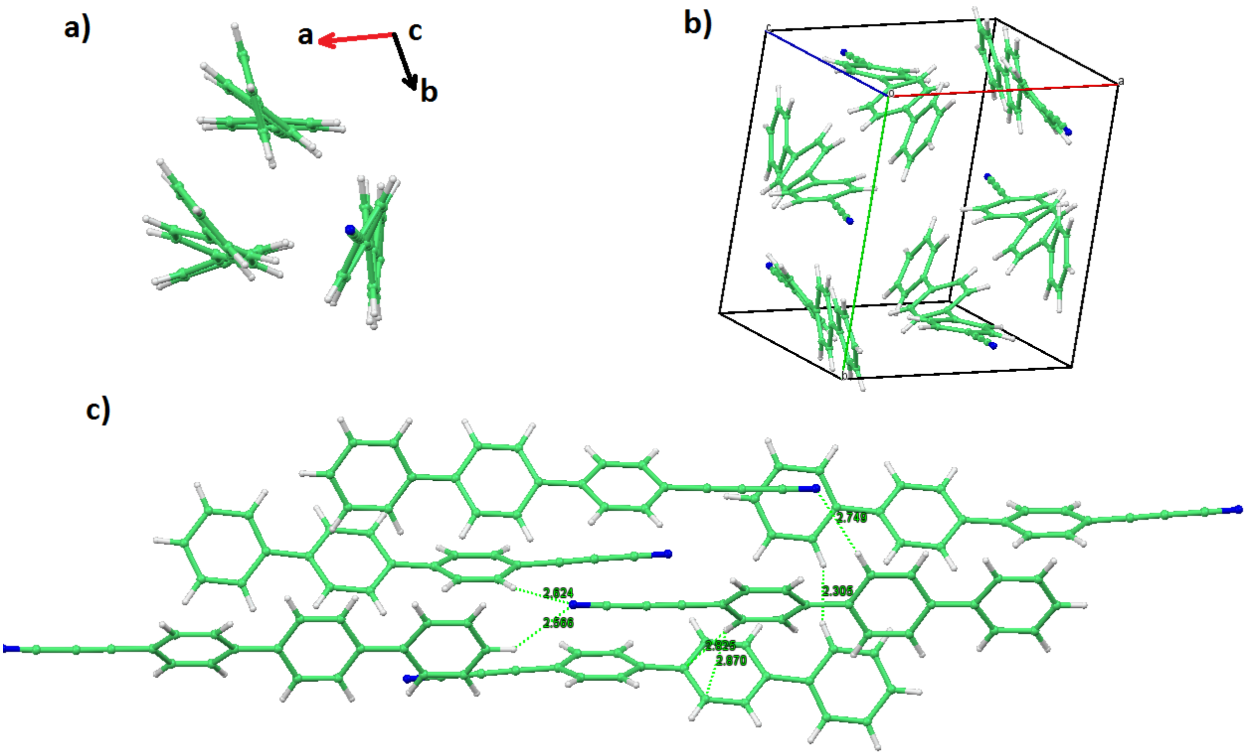

Figure S3: The crystal structure of compound **3** as obtained by XRD analysis. a,b) 3D visualization of the molecular conformation (view along direction *c*); c) Molecules arrange in parallel layers. Propiolonitrile groups acquire an antiparallel ordering motif. Two H bonds of the N···CH type are present. All distances are given in Å.

Table S3: Crystal data and structure refinement for **3**.

Compound	**3**
Empirical formula	$C_{21}H_{13}N$
Formula weight	279.32
Temperature/K	180.15
λ/Å	0.71073
Crystal system	triclinic
Space group	*P*-1
a/Å	9.816(2)
b/Å	12.229(2)
c/Å	19.558(4)

α/°	98.36(3)
β/°	101.77(3)
γ/°	102.50(3)
Volume/Å^3	2199.0(9)
Z	6
ρ_{calc} mg/mm^3	1.266
μ/mm^{-1}	0.073
F(000)	876.0
Crystal size/mm^3	0.43 × 0.39 × 0.06
2θ range for data collection/°	4.342–51.158
Reflections collected	15902
Independent reflections	8004 [R_{int} = 0.0950, R_{sigma} = 0.1090]
Data/restraints/parameters	8004/0/595
Goodness-of-fit on F^2	0.995
Final *R* indexes [*I* >= 2σ (*I*)]	R_1 = 0.0923, wR_2 = 0.2641
Final *R* indexes [all data]	R_1 = 0.1533, wR_2 = 0.3006
Largest diff. peak/hole / e Å^{-3}	0.58/−0.35

Table S4: Selected bond lengths (Å) in compound **3**.

N1- C1	1.144(6)	C10- C15	1.394(6)
C1- C2	1.362(6)	C11- C12	1.378(6)
C2- C3	1.203(6)	C12- C13	1.382(6)
C3- C4	1.413(6)	C13- C14	1.378(6)
C4- C5	1.369(6)	C13- C16	1.484(5)
C4- C9	1.389(6)	C14- C15	1.393(6)
C5- C6	1.373(6)	C16- C17	1.395(6)
C6- C7	1.384(5)	C16- C21	1.383(6)

C7- C8 1.390(6)	C17-C18 1.386(6)
C7- C10 1.483(5)	C18-C19 1.380(7)
C8- C9 1.375(6)	C19-C20 1.342(7)
C10-C11 1.365(6)	C20-C21 1.378(6)

References

[1] McAllister, G.D.; Wilfred, C.D.; Taylor, R.J. *Synlett* **2002**, 1291–1292.

[2] Sviridenko, F. B.; Stass, D. V.; Kobzeva, T. V.; Tretyakov, E. V.; Klyatskaya, S. V.; Mshvidobadze, E. V.; Vasilevsky, S. F.; Molin, Y. N. *J. Am. Chem. Soc.* **2004,** *126,* 2807–2819. doi:10.1021/ja037157m

[3] Schlickum, U.; Klappenberger, F.; Decker, R.; Zoppellaro, G.; Klyatskaya, S.; Ruben, M.; Kern, K.; Brune, H.; Barth, J. V. *J. Phys. Chem. C* **2010,** *114,* 15602–15606. doi:10.1021/jp104518h

[4] Klappenberger, F., Kühne, D., Marschall, M., Neppl, S., Krenner, W., Nefedov, A., Strunskus, T., Fink, K., Wöll, C., Klyatskaya, S., Fuhr, O., Ruben, M. and Barth, J. V. *Adv. Funct. Mater*. **2011**, 21, 1631–1642.

[5] Britton, D.; Young, V. G. *Acta Crystallogr., Sect. E: Struct. Rep. Online* **2003**, *59*, 1849.
